# A silent link between stillbirth and maternal anaemia: evidence from the fifth round of the National Family Health Survey

**DOI:** 10.1186/s12978-026-02378-x

**Published:** 2026-05-27

**Authors:** Mohammad Hammad, Mohammad Hifz Ur Rahman, Mohd Asfahan Nomani

**Affiliations:** 1 Manipal Tata Medical College, Manipal Academy of Higher Education, Manipal, India; 2 National University of Science and Technology, Sohar, Oman

**Keywords:** Stillbirth, Anaemia, Pregnancy Outcomes, NFHS, India

## Abstract

**Background:**

Stillbirth is a critical public health concern in India, with maternal anaemia being a significant contributing factor. Anaemia often resulting from a nutritional deficiciencies, has been linked to adverse pregnancy outcomes including stillbirth. This study aims to investigate the relationship between anaemia and the risk of stillbirth in India, incorporating data from the fifth wave of the National Family Health Survey.

**Methods:**

We utilized data from the fifth wave of the National Family Health Survey (NFHS) in India, utilizing information from 204,723 women 15–49 years of age who experienced a pregnancy in the five years preceding the survey. Calculations of stillbirth were performed via calendar data, which yielded reliable estimates. Descriptive statistics and results from bivariate analysis with chi-square tests have been reported to measure the prevalence. Furthermore, the study employed logistic regression to assess the relationship between stillbirth and maternal anaemia, adjusting for socioeconomic and maternal factors.

**Results:**

Stepwise logistic regression estimates revealed that women who have anaemia during pregnancy have a greater risk of stillbirth than non-anaemic women do, and this risk remains greater in the deduced models. We found that anaemic women have a stillbirth rate of 16.5 per 1000 total births, significantly higher than that of non-anaemic women (14.7 per 1000 total births).

**Conclusion:**

Our findings suggest that maternal anaemia increases the risk of stillbirth. Special attention is needed to reduce the prevalence of anaemia among women of reproductive age to decrease the burden of adverse pregnancy outcomes.

## Introduction

Stillbirth is a significant public health challenge, with far-reaching consequences for families and healthcare systems [1]. The World Health Organization defines stillbirth as foetal death occurring beyond the age of 28 weeks or greater; stillbirth is not only a devastating experience for parents inflicting emotional distress but also a key indicator of the overall quality and accessibility of maternal healthcare services [2, 3]. At 1.9 million stillbirths annually, the global burden of stillbirths continues to be a significant challenge [4]. Stillbirth remains a critical health issue in India, with 592,000 cases estimated annually, one-fourth of the global stillbirth burden. Notably, nearly 40% of stillbirths occur during labour, many of which can be averted through better quality healthcare during childbirth [5].

On the global front, the impact of stillbirths is significant, but there has been very little concern about the global health agenda in terms of maternal and child mortality [5–7]. This concern can be validated by examining the 2030 Agenda of Sustainable Development Goals, where no targets were made explicitly concerning stillbirth.

India has made remarkable progress in reducing stillbirth rates over the past twenty years, with rates decreasing from 29.6 per 1000 births in the year 2000 to 12.2 per 1000 births in 2021 [7]. Acknowledging stillbirths as persistent health challenges, India launched the India Newborn Action Plan (INAP) in 2014 with an ambition to reduce the stillbirth rate to less than 10 per 1000 births by the year 2030 [8].

Efforts have been made globally and nationally to address stillbirths, and it is crucial to recognize the interconnected nature of maternal health challenges. Among the various determinants of adverse pregnancy outcomes, maternal anaemia emerges as a critical and often overlooked factor. Its widespread prevalence, particularly in low- and middle-income countries such as India, poses a significant barrier to achieving targeted reductions in stillbirth rates.

Anaemia, a major public health concern, affects mainly young children, pregnant and postpartum women, and adolescent girls and women, especially in developing countries [9–11]. Anaemia, characterized by a reduced haemoglobin concentration, can have serious consequences, with an elevated risk of detrimental maternal and foetal outcomes, including stillbirth [12–14].

Globally, 40% of all children aged 6–59 months, 37% of pregnant women, and 30% of women aged 15–49 years are estimated to be affected by anaemia. In 2019, anaemia resulted in 50 million years of healthy life lost due to disability [15–17]. The greatest burden of anaemia is carried by low- and middle-income countries, particularly in populations living in rural settings, in poorer households and those who have received no formal education [15–17].

In India, the prevalence of anaemia among pregnant women is alarmingly high, with estimates during pregnancy ranging from 40% to 50% [18, 19]. The high prevalence of anaemia in India can be attributed to low dietary intake of iron and folic acid, poor bioavailability of iron, and chronic blood loss due to infections [10]. Anaemia during pregnancy can lead to complications such as haemorrhage, rupture of membranes, and reduced labour capacity, all of which can contribute to the risk of stillbirth [12].

To curb the high prevalence of anaemia, the Indian government, in 2018, under the National Health Mission, launched the Anaemia Mukt Bharat (AMB) Scheme, which aims to reduce the prevalence of anaemia across all age groups in the country, particularly women, children and adolescents. The program adopts a life-cycle approach with a focus on six target groups and includes interventions such as the supplementation of iron and folic acid, deworming, addressing behavioural changes, and performing tests to identify and treat cases of anaemia and taking into account non-nutritional causes. It also emphasizes convergence across various ministries for a holistic approach to anaemia reduction. The Anaemia Mukt Bharat Scheme aims to achieve a 3% annual reduction in anaemia prevalence, contributing to improved public health outcomes.

Globally, the inherent effects of anaemia have been irrefutable, although the distinctive impact within India’s disparate socioeconomic and cultural contexts remains untraversed. Most previous studies have been confined to localized or regional contexts, restricting broader applicability of their findings countrywide [20–22]. Furthermore, shifts in the policy environment, including the enactment of the Anaemia Mukt Bharat (AMB) initiative, may have altered the relationship between anaemia and pregnancy outcomes, reciprocating the necessity for fresh research with updated data.

A large, nationally representative cross-sectional survey conducted in India from 2019 to 2021, the National Family Health Survey-5 (NFHS-5), offers a unique opportunity to investigate the relationship between anaemia and stillbirth risk. Various covariates and robust mortality estimates allow the NFHS-5 to provide comprehensive analysis, taking into account a variety of sociodemographic, maternal and health factors that may affect pregnancy outcomes.

The findings of this study have the potential to inform targeted interventions and public health policies aimed at reducing the burden of anaemia and stillbirth in India. Timely and appropriate nutritional counselling, improved training and treatment, the promotion of effective monitoring and convergence, and improved access to high-quality health services may play key roles in reducing birth risk and protecting both mothers and foetuses. Additionally, this study is in line with the United Nations Sustainable Development Goals (SDGs), especially Goals 3 (Good Health and Well-Being) and 2 (Zero Hunger). These findings emphasize the broader relevance of sustainable development in influencing maternal and child health.

## Theoretical framework

The present study is grounded in several theoretical perspectives that provide a comprehensive framework for understanding the relationship between maternal anaemia and stillbirth. Anaemia is caused by several factors that significantly affect the oxygen supply in the human body, causing tiredness, weakness and shortness of breath [23]. Pregnant women who are anaemic face a number of pregnancy complications and adverse pregnancy outcomes [20].

Anaemia is the most common haematological abnormality among pregnant women. The primary reason for anaemia is the deficiency of iron as a micronutrient in the body, which is usually caused by poor dietary intake [24]. However, anaemia is not caused by merely one reason; multiple factors are responsible for it. Studies have reported that anaemia is significantly associated with parasitic infections [25], family history [26], and heavy menstrual bleeding among women of reproductive age [27].

Notably, several risk factors combine to influence stillbirth as an eventual pregnancy outcome. Prior studies have established that advanced maternal age [28], pregestational obesity [29], maternal exposure to alcohol [30] and indoor air pollution due to the use of unimproved sources for cooking [31] increase the risk of stillbirth. Pregestational morbidities such as diabetes, hypertensive disorders, and anaemia are important predictors of pregnancy complications and adverse birth outcomes [32, 33].

**Fig. 1:**
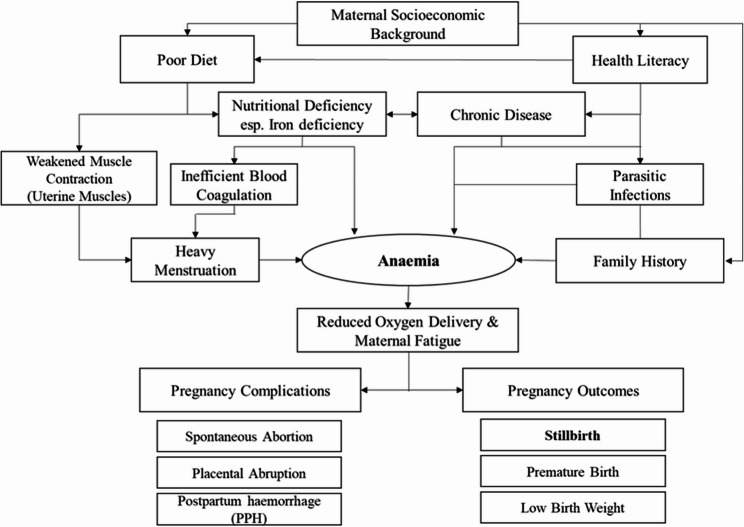
Conceptual Framework Source: Authors conceptualization

Iron deficiency anaemia causes reduced oxygen delivery to tissues, resulting in maternal fatigue, breathlessness, and even fainting [34]. Maternal anaemia is associated with pregnancy complications and adverse birth outcomes [35–37] among them, stillbirth is one of the major contributors. A retrospective cohort study in China revealed that severe anaemia during pregnancy is associated with an increased risk of placental abruption, preterm birth, postpartum haemorrhage (PPH), and foetal malformation [38]. (Fig. 1**)**

## Data and methodology

The study draws on individual-level data from the fifth round of the National Family Health Survey (NFHS), a nationally representative survey conducted throughout India between 2019 and 2021. The survey aims to collate information on a wide range of indicators, including demographic and socioeconomic characteristics, maternal and child health, reproductive health and family planning.

For this analysis, the Individual Recode file (IAIR7EFL) was utilized, which provides comprehensive data on women’s demographic characteristics, pregnancy histories, and household-related variables. The study included a sample of 204,723 women between the ages of 15 and 49 who had undergone at least one pregnancy in the five years prior to the survey and women currently pregnant at the time of the survey.

The primary outcome variable examined in this study was stillbirth, defined as the intrauterine demise of a foetus after 28 weeks of gestation. Calendar data were utilized for estimating stillbirths, as they provide more accurate and consistent estimates than conventional measurement approaches do. These data, sourced from individual records, include monthly records of pregnancy outcomes. In this dataset, a live birth is marked as ‘B’, pregnancy termination as ‘T’, and an ongoing pregnancy as ‘P’. For example, a sequence such as ‘PPPPPPB’ indicates a live birth, whereas ‘PPPPPT’ reflects a pregnancy termination. A sequence showing termination after seven months of pregnancy, such as ‘PPPPPPPT’, was classified as a stillbirth. This outcome variable was subjugated into two sections: 0 for ‘live birth’ and 1 for ‘stillbirth’. Cases of abortion and miscarriage were excluded from the estimation.

The key exposure variable assessed in this study was the prevalence of anaemia among women; the other covariates were included based on the literature survey and the conceptual framework based on it. Anaemia was measured based on levels of haemoglobin measured at the time of the survey. The cutoff levels were based on the official DHS biomarker manual and World Health Organization [39, 40]. For women who were not pregnant at the time of the survey, the cutoff was defined as Hb < 12.0 g/dL and likewise for women who were pregnant at the time the cutoff was defined as Hb < 11.0 g/dL. A single variable was then created with ‘0’ representing women who are ‘Not Anaemic’ and ‘1’ determining women who are ‘Anaemic’. The analysis also incorporated several sociodemographic variables, including age, educational attainment, the household wealth index, place of residence, and social group, as these elements significantly influence individuals’ access to essential services and resources. Additionally, maternal characteristics—such as the place of delivery, type of delivery, and frequency of antenatal care visits—were included in the analysis.

The selection of the covariates was guided by the conceptual framework, which incorporates both established risk factors and possible confounding variables influencing the link between anaemia and stillbirth. This framework considers a range of determinants operating at the individual, household, and community levels, all of which can influence pregnancy outcomes.

## Statistical analysis

To address the multifaceted sampling structure of the National Family Health Survey (NFHS), a comprehensive analytical strategy was employed. Initially, univariate analysis was used to describe the sample and examine background characteristics. Bivariate analysis with the chi-square test was subsequently conducted to evaluate the association between stillbirth and anaemia.

For the primary analysis, binary logistic regression was utilized in the stepwise approach by including various covariates stepwise to elucidate on the unadjusted as well as the overall odds of stillbirth by including the covariates in a stepwise approach.

The regression was performed using survey weights and incorporating the survey’s stratification and clustering design features. Sampling weights were included to correct for unequal probabilities of selection, thereby enhancing the representativeness of the estimates. Additionally, strata and cluster identifiers were added to account for intracluster correlation and prevent the undervaluation of standard errors. The analysis was carried out via the ‘svyset’ command. This methodological approach allowed the examination of the link between stillbirth and anaemia while simultaneously adjusting for confounding variables and including relevant sociodemographic and maternal covariates.

## Results

The study sample consisted of approximately 2 lakh women who were recently pregnant; among them, nearly 59% were anaemic. The majority of them (71.9%) were in the 21–30 years age group, and approximately 14% were under 20 years of age. Among the total sample of women, 71% live in rural areas, and approximately 20% do not have any formal education. In response to the question related to their recent delivery, 24% had caesarean delivery, and approximately 62% had delivery at public facilities, whereas 28% and 10% had delivery at private and other places, respectively. Among the total sample of women who were recently pregnant, 20% did not have antenatal care (ANC) visits, approximately 42% had 1 to 4 ANC visits, and approximately 37% had more than 4 ANC visits (Table 1).

**Table 1:** Background characteristics of the study sample of women aged 15–49 years in India 2019-21

Variables	Sample	weighted %
*Main Exposure variable*		
Whether having anaemia		
Not anaemic	82,690	41.2
Anaemic	114,334	58.8
*Background characteristics*		
Place of residence		
Urban	45,025	29.0
Rural	159,698	71.0
Highest Level of Education		
No formal education	41,978	19.6
Primary	25,351	11.8
Secondary	106,987	51.4
Higher	30,407	17.1
Wealth category		
Poor	97,669	43.2
Middle	40,229	19.6
Rich	66,825	37.2
Social category		
Scheduled caste	40,601	22.6
Scheduled tribe	40,702	9.8
Other backward class	77,335	42.9
General/ upper caste	46,085	24.8
*Maternal Health characteristics*		
Age of mother		
20 years & below	25,098	13.8
21–30 years	141,754	71.9
30 years & above	33,014	14.4
Place of Delivery		
Public	114,951	61.9
Private	40,280	28.2
Other	21,611	9.9
Antenatal visits		
No visits	41,703	20.1
1 to 4 visits	89,110	42.4
More than 4 visits	73,910	37.5
Had caesarean delivery		
Yes	37,759	24.0
No	139,083	76.0
N	204,723	100.0

The prevalence of stillbirth was significantly greater among anaemic women than among their non-anaemic counterparts (Table 2). Women of younger ages have a higher proportion of stillbirth; women aged 20 and younger have a prevalence of 1.65%, whereas women aged between 20 and 30 years and 30 + years have a prevalence of 1.27% and 0.96%, respectively.

**Table 2:** Prevalence of Stillbirth among recently pregnant women with respect to the background variables in India, 2019-21

Variables	Stillbirth	Weighted %	chi-sq *p*-value
*Main Exposure variable*			
Whether having anaemia			
Not anaemic	952	1.47	0.00*
Anaemic	1535	1.65	
*Background characteristics*			
Place of residence			
Urban	452	1.29	0.00*
Rural	2144	1.66	
Highest education			
No formal education	651	1.95	0.00*
Primary	356	1.69	
Secondary	1289	1.52	
Higher	300	1.13	
Wealth category			
Poor	1456	1.82	0.00*
Middle	474	1.56	
Rich	666	1.24	
Social category			
Scheduled caste	634	1.98	0.00*
Scheduled tribe	463	1.46	
Other backward class (OBC)	988	1.48	
General/ upper caste	511	1.37	
*Maternal Health characteristics*			
Age of mother			
20 years & below	318	1.65	0.00*
21–30 years	1493	1.27	
30 years & above	285	0.96	
Place of delivery			
Public	933	0.95	0.00*
Private	444	1.28	
Other	199	1.26	
Antenatal visits			
No visits	1123	4.04	0.00*
1 to 4 visits	858	1.14	
More than 4 visits	615	1.01	
Had caesarean delivery			
Yes	404	0.98	0.00*
No	1172	1.36	

It has been observed that stillbirth is more common among women from disadvantaged socioeconomic backgrounds. The proportion of women reporting stillbirth is higher in rural areas (1.66%) than in urban areas (1.29%). Women with no formal education had the highest prevalence of stillbirth (1.95%). Similarly, women lying in the poor wealth quintile have a higher prevalence (1.82%) of stillbirth, and with increasing economic prosperity (Middle: 1.56%; Rich: 1.24%), the prevalence of stillbirth decreases. In terms of social category, scheduled caste women have the highest prevalence of stillbirth, i.e., 1.96%, whereas only 1.37% of the upper caste pregnant women end up with stillbirth.

The bivariate analysis also suggested that women with no ANC visits had a greater prevalence of stillbirths (4.04%). Additionally, the proportion of stillbirths was lower among women delivering in public health facility (0.95%) than those at private hospitals or clinics (1.28%).

**Fig. 2:**
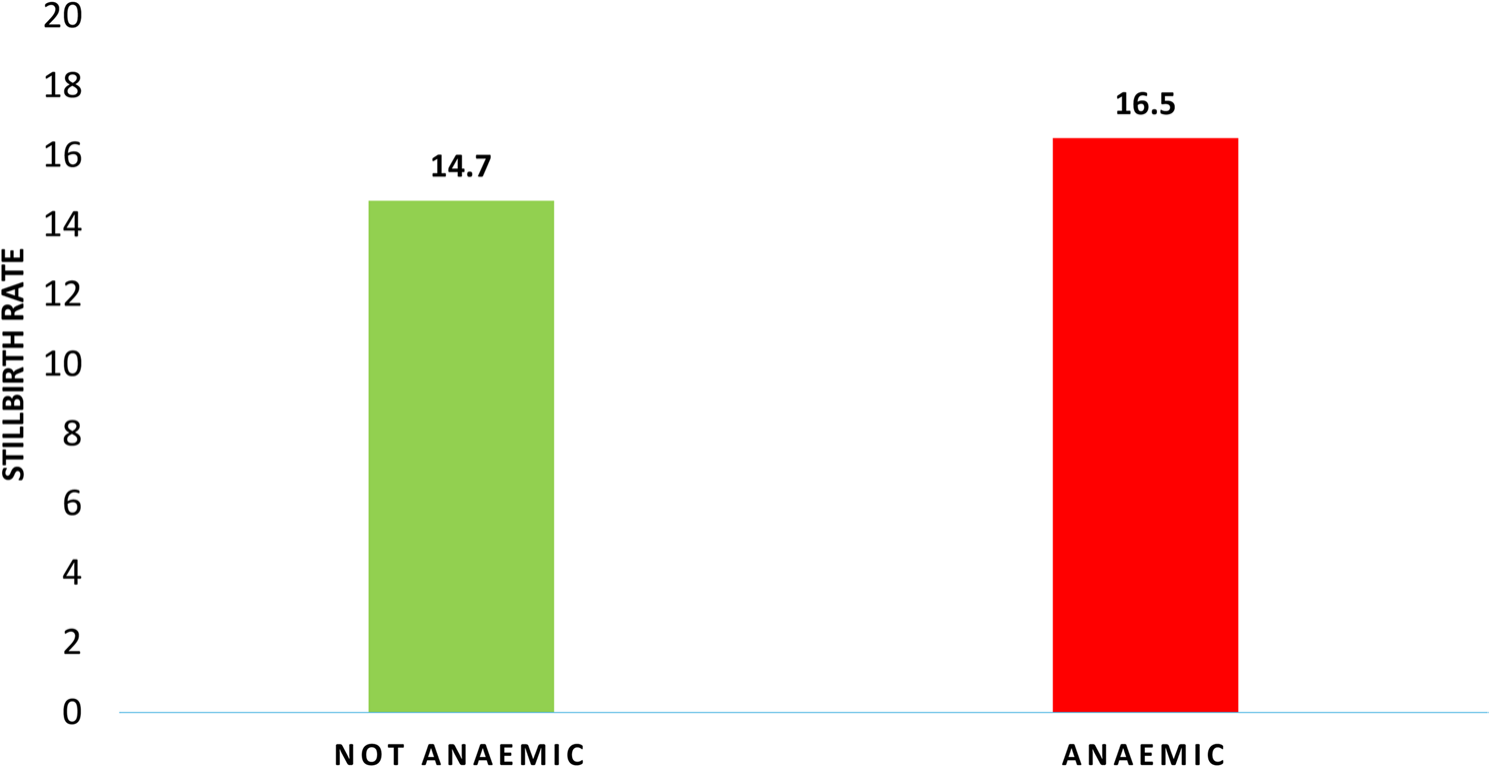
Stillbirth Rate (per 1000 total births) among anaemic and non-anaemic women

Figure 2 shows the stillbirth rate calculated per thousand total births. The stillbirth rate is clearly higher among anaemic women (16.5 per 1,000 total births) than among non-anaemic women (14.7 per 1,000 total births).

Table 3 presents the stepwise logistic regression results estimating the odds of stillbirth among women of reproductive age. Model 1 shows the unadjusted association, indicating that anaemic women have 12% higher odds of stillbirth compared to non-anaemic women (OR: 1.12; 95% CI: 1.01–1.25). Model 2 adjusts for maternal characteristics, with odds increasing to 25%. Model 3 further adjusts for socioeconomic characteristics of pregnant women, having and 19% higher odds of stillbirth. The variation in estimates across models reflects the influence of confounding factors; however, the positive association between anaemia and stillbirth persists after adjusting for these controls.

**Table 3:** Adjusted Odds of Stillbirth among recently pregnant women with respect to the background variables in India, 2019-21

Variables	Model-1^a^	Model-2^b^	Model-3^c^
	OR (95% CI)	AOR^d^ (95% CI)	AOR (95% CI)
*Main exposure variable*			
Having anaemia			
Not anaemic (ref.)	1.00	1.00	1.00
Anaemic	1.12* (1.01–1.25)	1.25* (1.09–1.45)	1.19* (1.04–1.38)
*Maternal Health characteristics*			
Age of woman			
20 years and below (ref.)	-	1.00	1.00
21–30 years	-	0.91 (0.74–1.11)	0.96 (0.78–1.18)
30 years & above	-	0.69* (0.53–0.91)	0.70* (0.53–0.93)
Place of delivery			
Public (ref.)	-.	1.00	1.00
Private	-	1.25* (1.06–1.49)	1.55* (1.28–1.86)
Other	-	1.39* (1.13–1.71)	1.21 (0.98–1.49)
Antenatal visits			
No Visits (ref.)	-	1.00	1.00
1 to 4 visits	-	1.12 (0.85–1.73)	1.27 (0.89–1.81)
More than 4 visits	-	1.04 (0.73–1.49)	1.23 (0.85–1.78)
Had caesarean delivery			
No (ref.)	-	1.00	1.00
Yes	-	1.40* (1.17–1.67)	1.54* (1.28–1.84)
*Background characteristics*			
Type of place of residence			
Urban (ref.)	-	-	1.00
Rural	-	-	1.19 (0.96–1.47)
Highest level of education			
No formal education (ref.)	-	-	1.00
Primary	-	-	0.87 (0.70–1.09)
Secondary	-	-	0.72* (0.61–0.87)
Higher	-	-	0.59* (0.44–0.78)
Wealth category			
Poor (ref.)	-	-	1.00
Middle	-	-	0.80* (0.67–0.95)
Rich	-	-	0.65* (0.52–0.81)
Social category			
General/ Upper caste (ref.)	-	-	1.00
Scheduled caste	-	-	1.31* (1.05–1.62)
Scheduled tribe	-	-	0.77* (0.60–0.99)
Other backward classes (OBC)	-	-	0.97* (0.81–1.18)

a Unadjusted model

b Considers maternal characteristics

c Considers maternal as well as background characteristics

d Adjusted Odds Ratio

## Discussion

The nationwide analysis underscores the notable influence of anaemia on negative pregnancy outcomes, especially stillbirth. In India, women diagnosed with anaemia are observed to have 19% greater odds of stillbirth than non-anaemic women. These results are consistent with earlier studies that established a heightened risk of adverse birth outcomes, such as stillbirth, which are linked to conditions such as iron deficiency anaemia, sickle cell anaemia, hookworm infection, and malaria [20, 41].

The pathophysiological mechanisms leading to stillbirth include reduced oxygen-carrying capacity of the blood, reduced placental perfusion and increased risk of intrauterine growth restriction (IUGR), which can lead to foetal hypoxia and subsequent stillbirth. Additionally, iron deficiency anaemia, the most prevalent form, has been associated with an increased likelihood of maternal infections, preterm delivery, and obstetric haemorrhages, all of which can increase the risk of stillbirth [42]. Anaemia can exacerbate other pregnancy complications, such as preeclampsia and gestational hypertension, further increasing the risk of adverse outcomes, particularly stillbirth [43].

The findings of this study also align with global research. A meta-analysis by Rahman et al. [42] reported that maternal anaemia increased the risk of stillbirth by 29% [44]. Similarly, studies in sub-Saharan Africa and South Asia have consistently identified anaemia as a significant predictor of stillbirth [45]. These findings underscore the universal importance of addressing maternal anaemia to improve pregnancy outcomes, particularly in low- and middle-income countries.

The implementation of the Anaemia Mukt Bharat (AMB) initiative in 2018 represents a concerted effort to address anaemia in the country through strategies such as intensified iron supplementation, dietary counselling and behavioural change communication. However, the persistence of high anaemia prevalence suggests that additional measures are needed. Strengthening health system capacity, improving the coverage of antenatal care services, and addressing social determinants of health, such as poverty and food insecurity, are essential for reducing the dual burden of anaemia and stillbirth in India.

An inverse association between maternal education and the likelihood of emphasizing the importance of women’s empowerment and educational attainment. Women with higher levels of education often possess better knowledge of health-related risks, improved access to medical services, and increased capacity to make informed decisions about household management and resource utilization [46]. Expanding educational access for women, particularly in rural and marginalized populations, could play a critical role in reducing stillbirth incidence.

The study’s insights regarding the place of delivery highlight the essential contribution of quality healthcare during pregnancy. Compared with their corresponding reference categories, women who give birth in private medical institutions are more likely to stillbirth [47]. While the dataset does not directly reveal the causes of these associations, it may reflect broader issues such as socioeconomic inequality, limited access, or variations in care quality across healthcare sectors.

Additionally, the findings indicated a greater stillbirth risk among women who underwent caesarean delivery. Although a direct causal link cannot be inferred from the analysis, it is conceivable that maternal or foetal complications requiring surgical intervention may partly explain the increased risk [48]. In low-resource environments, caesarean births may also be associated with increased risks due to inadequate infrastructure or insufficient postoperative care [49]. There is also a possibility of reverse causality, as caesarean delivery is often performed in response to pregnancy complications.

In addition to the direct effects of anaemia, the analysis revealed considerable disparities in stillbirth risk linked to sociodemographic and maternal characteristics. Women residing in economically disadvantaged households, those with minimal educational attainment, and individuals belonging to socially marginalized communities were observed to face a greater risk of stillbirth. These outcomes underscore the urgent need for focused strategies and policy-level interventions to address the root causes of socioeconomic inequality and limited healthcare access that contribute to negative pregnancy outcomes.

The findings further emphasize the role of high-quality maternal healthcare during pregnancy, as indicated by the elevated risk of stillbirth among women delivering in private healthcare settings. Those who have fewer antenatal care visits may miss opportunities for early detection of pregnancy-related complications, preventive care, and timely medical interventions. Strengthening access to comprehensive prenatal services and encouraging institutional deliveries in adequately equipped healthcare centres could serve as critical measures to mitigate the incidence of stillbirth.

A key strength of this study is its broad national coverage, utilizing data from the fifth round of the National Family Health Survey (NFHS-5), which is a large-scale, nationally representative dataset. This extensive representation allows the findings to be applicable across India’s varied socioeconomic and cultural landscape. Additionally, the study benefits from the use of meticulous sampling strategies and high-quality data collection protocols, which contribute to the credibility and robustness of the results.

While the study established a relationship between anaemia and adverse pregnancy outcomes, its cross-sectional design limits the ability to draw causal conclusions. To better understand the directionality and underlying mechanisms, longitudinal research is warranted. The reliance on self-reported data introduces the potential for recall bias or underreporting. Moreover, the calculation of stillbirths did not account for miscarriages and abortions. The analysis also lacked detailed information on the timing and duration of anaemia—such as whether it occurred prior to pregnancy or during specific trimesters—which may affect the strength of the associations observed. Although the magnitude of the association between stillbirth and anaemia is modest, even small increases in risk can have significant public health implications in a country with a high prevalence of maternal anaemia and a large population. Additionally, unmeasured factors such as maternal nutrition, environmental conditions, and accessibility to prenatal care could influence the results and merit further investigation.

Further, a primary limitation of this study is that the National Family Health Survey (NFHS), which measures biomarkers such as haemoglobin at the time of the interview rather than at the time of the pregnancy. This creates a temporal gap between the exposure (anaemia) and the outcome (stillbirth). However, in the Indian context, maternal anaemia is largely driven by chronic nutritional deficiencies and structural socio-economic factors that remain relatively stable over a woman’s reproductive life course [50]. Given the ‘steady-state’ nature of nutritional status in high-burden settings, current haemoglobin levels likely serve as a reliable proxy for a woman’s physiological environment during her previous pregnancies. While we cannot definitively establish a causal temporal sequence, these findings identify a critical association between long-term maternal nutritional vulnerability and adverse obstetric outcomes. Future longitudinal studies are required to confirm the precise timing of haematological changes and their direct impact on foetal survival.

Despite these limitations, this study enhances our understanding of the complex relationship between anaemia and adverse pregnancy outcomes in India. The association between anaemia and stillbirth after adjusting for covariates highlights the urgent need to address anaemia through improved nutrition and health care interventions and to address the preexisting socioeconomic and environmental conditions contributing to the condition.

## Conclusion

This study provides evidence of the link between anaemia and stillbirth. This presents the prospect of new and novel mechanisms through which anaemia may affect stillbirth risk. This study suggests that proper attention must be given to decreasing the prevalence of maternal anaemia to reduce the risk of stillbirth in India. Government schemes such as ‘Anaemia Mukt Bharat’ should prioritize the beneficiary group of pregnant women and lactating mothers to combat the intergenerational flow of anaemia. Additionally, the Pradhan Mantri Surakshit Matritva Abhiyan for quality ANC checkups needs to be implemented more stringently.

## Data Availability

The data that support the findings of this study are available upon request. The dataset used in the study is available in the public domain and can be accessed on a request from the DHS at [https://dhsprogram.com/Data/](https:/dhsprogram.com/Data) . The dataset and materials used in this study are available upon request from the corresponding author.
